# Coat Color-Facilitated Efficient Generation and Analysis of a Mouse Model of Down Syndrome Triplicated for All Human Chromosome 21 Orthologous Regions

**DOI:** 10.3390/genes12081215

**Published:** 2021-08-06

**Authors:** Yichen Li, Zhuo Xing, Tao Yu, Annie Pao, Marcel Daadi, Y. Eugene Yu

**Affiliations:** 1 The Children’s Guild Foundation Down Syndrome Research Program, Genetics and Genomics Program and Department of Cancer Genetics and Genomics, Roswell Park Comprehensive Cancer Center, Buffalo, NY 14263, USA; yichen.li@roswellpark.org (Y.L.); zhuo.xing@roswellpark.org (Z.X.); taoyuwd@hotmail.com (T.Y.); annie.pao@roswellpark.org (A.P.); 2 Regenerative Medicine and Aging Unit, Texas Biomedical Research Institute, Long School of Medicine, University of Texas Health Science Center at San Antonio, San Antonio, TX 78245, USA; mdaadi@txbiomed.org; 3 Genetics, Genomics and Bioinformatics Program, State University of New York at Buffalo, Buffalo, NY 14203, USA

**Keywords:** Down syndrome, mouse models, coat colors, cognitive function

## Abstract

Down syndrome (DS) is one of the most complex genetic disorders in humans and a leading genetic cause of developmental delays and intellectual disabilities. The mouse remains an essential model organism in DS research because human chromosome 21 (Hsa21) is orthologously conserved with three regions in the mouse genome. Recent studies have revealed complex interactions among different triplicated genomic regions and Hsa21 gene orthologs that underlie major DS phenotypes. Because we do not know conclusively which triplicated genes are indispensable in such interactions for a specific phenotype, it is desirable that all evolutionarily conserved Hsa21 gene orthologs are triplicated in a complete model. For this reason, the Dp(10)1Yey/+;Dp(16)1Yey/+;Dp(17)1Yey/+ mouse is the most complete model of DS to reflect gene dosage effects because it is the only mutant triplicated for all Hsa21 orthologous regions. Recently, several groups have expressed concerns that efforts needed to generate the triple compound model would be so overwhelming that it may be impractical to take advantage of its unique strength. To alleviate these concerns, we developed a strategy to drastically improve the efficiency of generating the triple compound model with the aid of a targeted coat color, and the results confirmed that the mutant mice generated via this approach exhibited cognitive deficits.

## 1. Introduction

**Figure 1:**
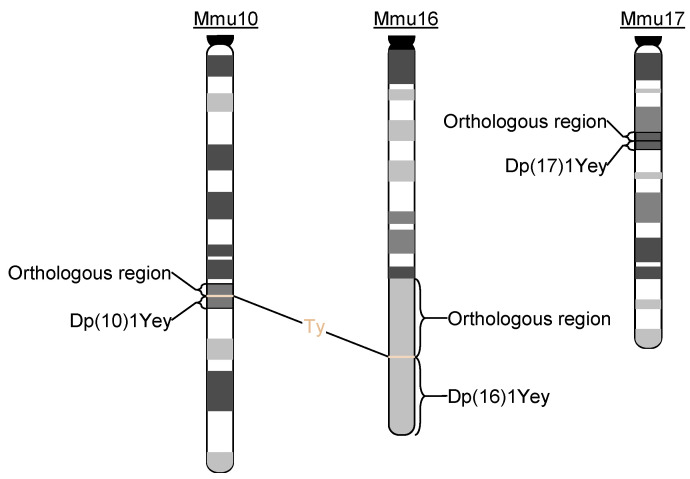
Presence of the *Ty* transgene at the breakpoints of Dp(10)1Yey and Dp(16)1Yey but not Dp(17)1Yey.

Human trisomy 21, which is associated with Down syndrome (DS), occurs in one in approximately 800 live births [1]. This chromosomal rearrangement leads to a constellation of phenotypes, and its effects on the nervous system are particularly problematic because the changes lead to developmental delays and intellectual disabilities with very high penetrance; notably, trisomy 21 is the most common chromosomal cause of developmental intellectual disabilities in humans [2,3]. Given the high degree of evolutionary conservation between humans and mice at the genomic and phenotypic levels, the mouse has remained the most extensively investigated model organism for DS (Figure 1). Considered by some investigators as a contiguous gene syndrome [4], the earliest group of genomic mouse models for DS included mouse trisomy 16, Ts65Dn, and Ts1Cje [5,6,7,8]. The mouse trisomy 16 model was developed based on the orthologous conservation between a part of human chromosome 21 (Hsa21) and the distal portion of mouse chromosome 16 (Mmu16) (www.ensembl.org, accessed on 20 June 2021). However, there are some major deficiencies associated with the mouse trisomy 16 model; namely, approximately 76.5% of Mmu16 is not orthologous to Hsa21 and none of the mutant mice can survive postnatally [8]. In contrast, Ts65Dn mice are viable postnatally, and these mice carry marker chromosome Ts(17^16^)65Dn, an unbalanced derivative of a balanced chromosomal translocation between Mmu16 and Mmu17. This extra chromosome spans the genomic region between *Mrpl39* and the telomere on Mmu16, which is orthologous to a region on Hsa21 and contains approximately 100 Hsa21 gene orthologs. Ts(17^16^)65Dn also contains a subcentromeric region of Mmu17 [9,10]. Therefore, Ts65Dn is not a complete mouse model of DS based on gene dosages. Ts1Cje is triplicated for a smaller Hsa21 orthologous region than that of Ts65Dn, and this region ranges from *Sod1* to the telomere on Mmu16 [7]. The second group of genomic mouse models developed were transchromosomal models [11,12]. Tc1, a transchromosomal mouse strain, represented an important model following the first group, and these mice carry a Hsa21-based transchromosome [12]. However, that transchromosome misses more than 50 Hsa21 genes because of the presence of several deletions [11,13]. In addition, Tc1 is a mosaic model so the transchromosome in Tc1 is not present in many cells in a mutant mouse and different Tc1 mutant mice have different levels of mosaicisms [13,14]. Recently, a new transchromosomal model that does not appear to be mosaic has been reported, but the transchromosome in TcMAC21 still misses 14 Hsa21 genes because of the presence of several deletions [11]. The third group of models developed involved duplication mice engineering primarily with mouse embryonic stem (ES) cell technology [15,16,17,18,19,20]. Among these, Dp(10)1Yey/+, Dp(16)1Yey/+, and Dp(17)1Yey/+, abbreviated hereafter as Dp(10)1Yey, Dp(16)1Yey, and Dp(17)1Yey, respectively, span the entire Hsa21 orthologous regions on Mmu10, Mmu16, and Mmu17, respectively.

Studies on DS, including mouse-based studies, have revealed that there are complex interactions among triplicated genomic regions and Hsa21 gene orthologs, which are causally associated with DS phenotypes [18,21,22,23]. To mimic all the evolutionarily conserved gene interactions, it is necessary that all Hsa21 gene orthologs are triplicated in a model. For these reasons, the Dp(10)1Yey;Dp(16)1Yey;Dp(17)1Yey model has a unique advantage. However, it has been suggested that the generation of this triple compound mutant would be a daunting task that could be impractical to pursue [2,11,24]. In this report, we dispute this suggestion by describing an efficient coat color-facilitated strategy for generating this important compound mutant. The results of behavioral tests confirmed that these mutant mice exhibited developmental cognitive deficits.

## 2. Materials and Methods

### 2.1. Mice

Dp(10)1Yey, Dp16)1Yey, and Dp(17)1Yey mice were maintained in a 129Sv background [16,20]. Individual mouse mutants were backcrossed with albino B6 mice (C57B6/J-*Tyr*^c-Brd^) for two or three generations. For the identification of Dp(10)1Yey, specific polymerase chain reaction (PCR) primers 5′-AGC CGG CGA ACG TGG CGA GAA A-3′ and 5′-AAG GCC TGC TGC CAA GCC ATC AG-3′ were used. For the identification of Dp(16)1Yey, specific PCR primers 5′-CTG CCA GCC ACT CTA GCT CT-3′ and 5′-AAT TTC TGT GGG GCA AAA TG-3′ were used. For the identification of Dp(17)1Yey, specific PCR primers 5′-GCC AGC AAC GCG GCC TTT TTA CG-3′ and 5′-CAT TGG GGA GCC AGG GCT GAT GGT-3′ were used. The mutant mice and wild-type control mice were maintained at a temperature- and humidity-controlled animal facility with a 12:12 h light/dark cycle, and the mice had ad libitum access to food and water. All mouse experiments were approved by the Institute Animal Care and Use Committee.

### 2.2. Open Field Test

The mice were placed in an open field arena and tracked for 10 min. The arena was a transparent plastic box with the following dimensions: 40 (L) × 40 (W) × 40 cm (H); the box was divided into 25 zones, and the central nine zones were considered as the center. The experimental data were recorded and analyzed with an HVS Image All in One Tracking 2019 system (HVS Image Ltd., Twickenham, Middlesex, UK). The total distance traveled (cm), average velocity (cm/s), percentage of zones used, and percentage of time spent in the center were analyzed for each genotype.

### 2.3. T-Maze Spontaneous Alternation Test

To examine hippocampal function, continuous spontaneous alternation tests were performed based on the protocol of Deacon and Rawlins [25]. The T-shape maze was made of opaque acrylic (Plexiglas) with a start arm (90 × 10 × 20 cm) as well as left and right arms (37 × 10 × 20 cm for each arm). A sliding door was placed in the start arm to create a holding place that was separated from the rest of the maze, while a divider panel (20 × 20 cm), which extended 10 cm into the start arm, was centered in the middle of the “T” between the left and the right arms. At the beginning of the test, a mouse was placed into the start arm. After 5 min of confined time, the slide door was opened, and the mouse had free access to the rest of the maze for 10 min. Any entry into one of the lateral arms was recorded, and entry into an opposite arm was defined as an alternation. Only a valid entry, when the whole body of the mouse including its tail was inside the lateral arm, was counted. The maze was cleaned with 10% ethanol in between tests with different mice. The percentage of time the mouse alternated between left and right arms over the total number of entries was defined as alternation performance.

### 2.4. Nesting Test

To further test hippocampus-associated function in the mice, nesting tests were performed based on the protocol of Deacon [26]. Approximately 1 h before the dark phase, mice were separated into individual testing cages that contained corn cob bedding. Then, a 2-g pre-weighed pressed cotton square (Nestlet) was placed into each testing cage. After the 12-h dark phase, mice were returned to their home cages. The nests were scored, and the unused Nestlet was weighted. We used the following scoring system: 0 = Nestlet was not noticeably used; 1 = Nestlet was slightly used but not gathered; 2 = Nestlet was partly used and gathered, but there was no nest formed; 3 = Nestlet was partly used, and a flat nest was formed; 4 = Nestlet was mostly used to form a nest, but noticeable Nestlet remained; 5 = Nestlet was totally torn up to form a perfect nest, and no Nestlet remained.

### 2.5. Contextual Fear-Conditioning Test

The contextual fear-conditioning tests were performed using the Fear-Conditioning Video Tracking System (Med Associates Inc., St. Albans, VT, USA). The test chamber floor was a grid of stainless-steel rods connected to an electric shock generator. A video camera was mounted in the front of the chamber. A ceiling light illuminated the interior chamber through the transparent ceiling. Each mouse was allowed 2 min to explore the chamber as baseline activity before conditioning. Then, a foot shock (1 mA scrambled) with a duration of 2 s was delivered, which was controlled by Video Freeze Software V.1.8 (Med Associates Inc., St. Albans, VT, USA). After another 30 s, the mouse was removed from the test chamber. Each mouse was returned to the test chamber after 24 h and again after 72 h and monitored for 3 min with no foot shock. The Video Freeze Software recorded the freezing behavior automatically. Mean freezing time during the contextual exposure was calculated as a measure of contextual learning.

### 2.6. Foot-Shock Sensitivity Test

To ensure accurate interpretations of the fear-conditioning test data, foot-shock sensitivity tests were performed in the test chamber. During these tests, each mouse was placed into the chamber and subjected to a series of foot shocks delivered every 10 s starting at 0.05 mA, with a 0.05 mA increment between each shock. The minimal level of current needed to elicit flinching, vocalizing, or running was recorded.

### 2.7. Morris Water Maze Test

Hippocampal-dependent spatial memory was analyzed by using a standard Morris water maze test [27,28,29]. A circular pool (152 cm in diameter) was used, and the water had a temperature of 25 ± 1 °C. The experimental data were recorded and analyzed with the HVS Image All in One Tracking 2019 system (HVS Image Ltd., Twickenham, Middlesex, UK). Mice were trained with the following sequence of trials: visible platform, hidden platform, probe test, reversal platform, and reversal probe test. On day 0, visible platform trials were carried out with four trials for each mouse. In each trial, the mouse started from one of four start points (north, east, south, west) and swam for a maximum of 90 s to reach the platform. Once on the platform, the mouse was allowed to recover for 10 s before being gently removed from the pool. On days 1–7, the hidden platform trials were carried out, and each mouse was used in four trials each day. Data on the latency (time to find the platform), path-length (travel distance), and swimming speed were collected. On day 8, a probe test was administrated without the platform. Each mouse had 60 s to search the pool, and the percentages of time spent in different quadrants were recorded. On days 9–13, the reversal platform trials were carried out as previously described but the platform was hidden at the opposite quadrant. On day 14, the reversal probe test was administrated without the platform.

### 2.8. Sample Size and Statistical Methods

For the phenotypic analysis, five male and six female Dp(10)1Yey;Dp(16)1Yey;Dp(17)1Yey mice and six male and seven female wild-type control mice at 2–4 months of age were used, and these mice were identified via the coat color-facilitated strategy. The data from the T-maze spontaneous alternation tests, nesting tests, Morris water maze tests, contextual fear-conditioning tests, and foot-shock sensitivity tests, as well as body weight and body length measurements, were subjected to a one-way analysis of variance (ANOVA) between genotypes. The ANOVA did not detect any effects of sex in all the phenotypes, including the behavioral phenotypes, so the data from male mice and female mice were pooled and analyzed together for these experiments. All values reported in the text and figures were expressed as the means ± standard error of the mean (SEM).

## 3. Results

### 3.1. Efficient Generation of Dp(10)1Yey;Dp(16)1Yey;Dp(17)1Yey Mice Facilitated by Coat Colors

Mouse tyrosinase minigene (*Ty*) driven by its own regulatory element was a coat color marker gene in the targeting vectors used in engineering of the three Dp mutants, namely, Dp(10)1Yey, Dp(16)1Yey, and Dp(17)1Yey [16,20]. Because these targeting vectors were integrated into the genome of mouse ES cells via insertion events during gene targeting, all genetic elements of the targeting vectors were inserted into the targeted alleles, including *Ty* [16,20]. As a consequence, *Ty* is co-segregated with Dp(10)1Yey and Dp(16)1Yey in mutant mice (Figure 1) [16,20]. However, the presence or absence of this coat color was not our original concern when we designed our strategy to develop these mouse mutants. Therefore, *Ty* is not co-segregated with Dp(17)1Yey, but is with its reciprocal deletion, Df(17)1Yey [30].

**Figure 2:**
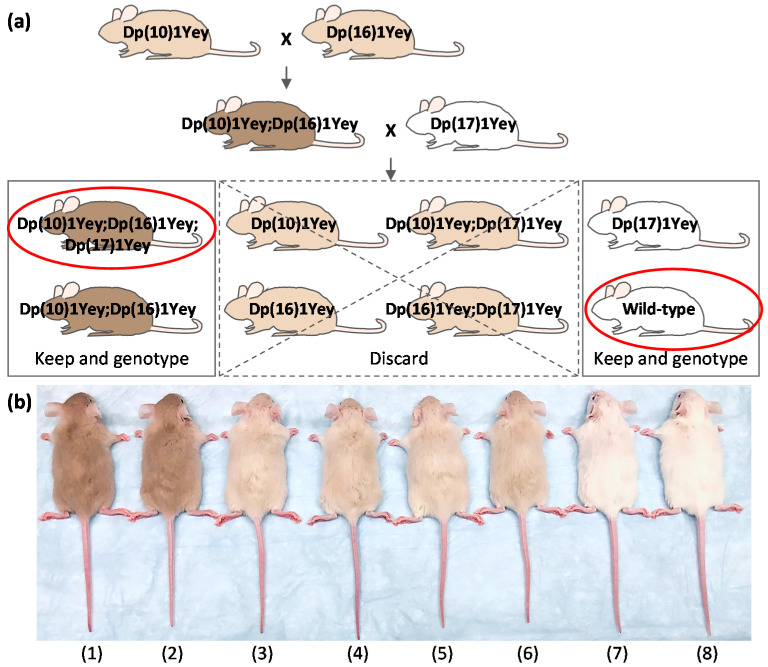
Coat color-facilitated strategy to identify the triple compound model of Down syndrome (DS). (**a**) A Dp(10)1Yey mouse with a cream coat color is crossed to a Dp(16)1Yey mouse with a cream coat color to produce a Dp(10)1Yey;Dp(16)1Yey mouse with a tan coat color. Further crossing of a Dp(10)1Yey;Dp(16)1Yey mouse and a Dp(17)1Yey mouse with a white coat color yields progeny with eight different genotypes. Among them, the ones with a cream coat color can be discarded because none of them carry the triple duplications. A single polymerase chain reaction (PCR) using the primers specific for Dp(17)1Yey can be used to identify the triple compound mutant mice and the wild-type control mice. (**b**) The relationship between the genotypes and coat colors among the progeny generated from the cross between Dp(10)1Yey;Dp(16)1Yey mice and Dp(17)1Yey mice. 1: Dp(10)1Yey;Dp(16)1Yey;Dp(17)1Yey, tan. 2: Dp(16)1Yey;Dp(17)1Yey, tan. 3: Dp(10)1Yey;Dp(17)1Yey, cream. 4: Dp(16)1Yey;Dp(17)1Yey, cream. 5: Dp(10)1Yey, cream. 6: Dp(16)1Yey, cream. 7: Dp(17)1Yey, white. 8: wild-type control, white. The mice in the photograph are four and a half weeks old.

Because of the growing concerns over the possible difficulties associated with the generation of the triple duplication mice as expressed by several groups [2,11,24], we decided to examine the efficiency of generating these mice with the aid of coat colors. After backcrossing to albino B6 mice, one copy of the *Ty* minigene led to a cream coat color in Dp(10)1Yey mice and Dp(16)1Yey mice (Figure 2b). To generate the triple duplication mice, we first crossed Dp(10)1Yey females with Dp(16)1Yey males because Dp(16)1Yey females showed an impaired ability to raise their pups. Dp(10)1Yey;Dp(16)1Yey mice were identified by the tan coat color resulting from the presence of two copies of *Ty* (Figure 2b), and the identifications were confirmed by PCR-based genotyping. Afterward, Dp(10)1Yey;Dp(16)1Yey mice were crossed to Dp(17)1Yey mice. The progeny from such a cross carried eight different genotypes and had three different coat colors (Figure 2).

The progeny with a tan coat color carried the genotype of Dp(10)1Yey;Dp(16)1Yey;Dp(17)1Yey or Dp(10)1Yey;Dp(16)1Yey. The progeny with a cream coat color carried the genotype of Dp(10)1Yey;Dp(17)1Yey, Dp(16)1Yey;Dp(17)1Yey, Dp(10)1Yey, or Dp(16)1Yey. The progeny with a white coat color carried the genotype of Dp(17)1Yey or a wild-type genotype. Therefore, to identify the triple compound mutant mice and their wild-type control mice, all the progeny with a cream coat color can be discarded because none of these carries triple duplications (Figure 2a). To identify the triple compound mice among the progeny with a tan coat color, we can perform a single PCR test using the primers specific for Dp(17)1Yey to distinguish Dp(10)1Yey;Dp(16)1Yey;Dp(17)1Yey mice and Dp(10)1Yey;Dp(16)1Yey mice. To identify the wild-type control mice among the progeny with a white coat color, we can perform a single PCR test using the primers specific for Dp(17)1Yey to distinguish Dp(17)1Yey mice and wild-type control mice. In one such breeding experiment, we identified 23 triple compound mutant mice and 31 wild-type litters, as well as the progeny with all other genotypes (Table 1). Their genotypes were confirmed by using the primers for identifying Dp(10)1Yey, Dp(16)1Yey, and Dp(17)1Yey (Table 1). These PCR-based genotyping results were matched with the coat colors, as shown in Figure 2, without exception.

Based on the above results, approximately 48.4% of the progeny do not need PCR-based genotyping because they have a cream coat color and thus do not carry the triple compound duplications (Figure 2, Table 1). For the remaining progeny (approximately 51.6%), we only need to perform a single PCR test for each one to identify the triple duplication mice and their wild-type control mice. In summary, by using this approach, approximately 82.8% of the genotyping efforts can be eliminated. The remaining 17.2% of the genotyping effort is sufficient for the identification of the triple compound mutant mice and their wild-type control mice. Therefore, DNA-based genotyping is about 581.4% more effective with the coat color-facilitated strategy than that without the proposed strategy for the identification of the triple compound model and their wild-type control mice.

### 3.2. Dp(10)1Yey;Dp(16)1Yey;Dp(17)1Yey Mice Identified via the Coat Color-Facilitated Strategy Exhibit Cognitive Deficits

**Figure 3:**
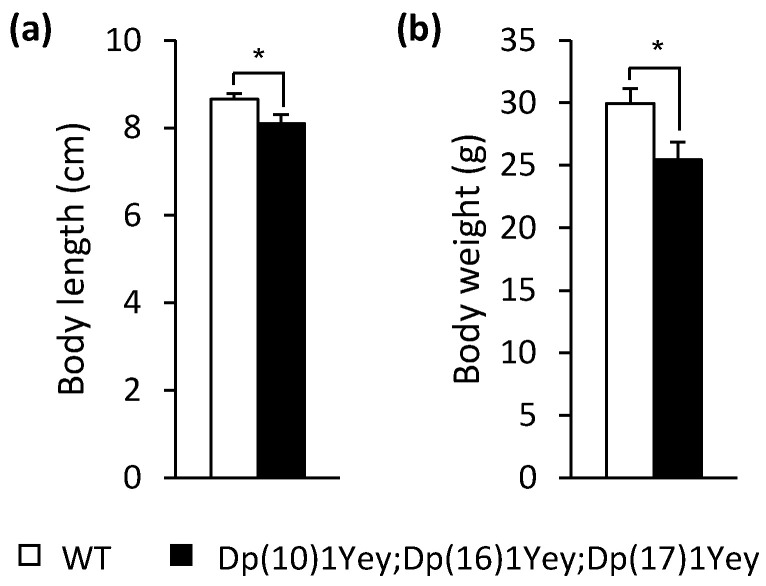
Body length and weight: (**a**) The mean nose-to-anus body length of Dp(10)1Yey;Dp(16)1Yey;Dp(17)1Yey mice was shorter than that of the wild-type control mice (*p* = 0.022). (**b**) The mean body weight of Dp(10)1Yey;Dp(16)1Yey;Dp(17)1Yey mice was smaller than that of the wild-type control mice (*p* = 0.023). *, *p* < 0.05.

**Figure 4:**
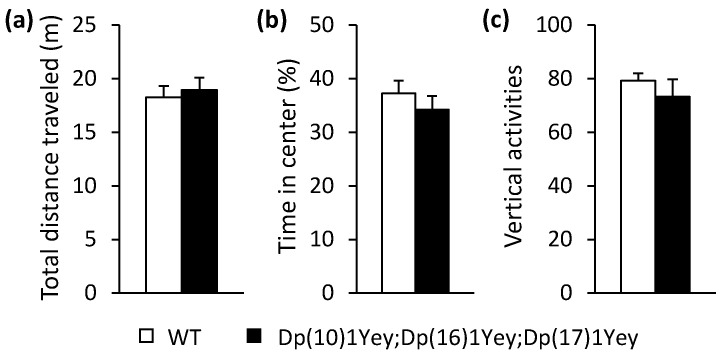
Open field test: Dp(10)1Yey;Dp(16)1Yey;Dp(17)1Yey and the wild-type control mice were placed in the open field arena for 10 min and their activities were recorded. There were no significant differences between these two groups of mice in terms of the total path traveled (**a**) (*p* = 0.67), time spent in the center arena (**b**) (*p* = 0.39), or total times of rearing (**c**) (*p* = 0.38).

Using the above coat color-facilitated strategy, we generated the triple compound model with a tan coat color and albino wild-type control mice. At 2–4 months of age, Dp(10)1Yey;Dp(16)1Yey;Dp(17)1Yey mice had a normal appearance. As indicated by the nose-to-anus body lengths and body weights, the triple compound mutant mice were smaller than the wild-type control mice (*p* < 0.05) (Figure 3). Because the presence of an exogenous coat color transgene in the albino background may alter vision and other phenotypic features, including those associated with behaviors [31,32,33,34,35,36,37,38], we next performed a battery of behavioral tests (as described in the Materials and Methods) on these two groups of mice at 2–4 months of age. In open field tests, we found no difference between the two groups in terms of the general activity level, gross locomotor activity, and exploratory habits (*p* > 0.05) (Figure 4).

**Figure 5:**
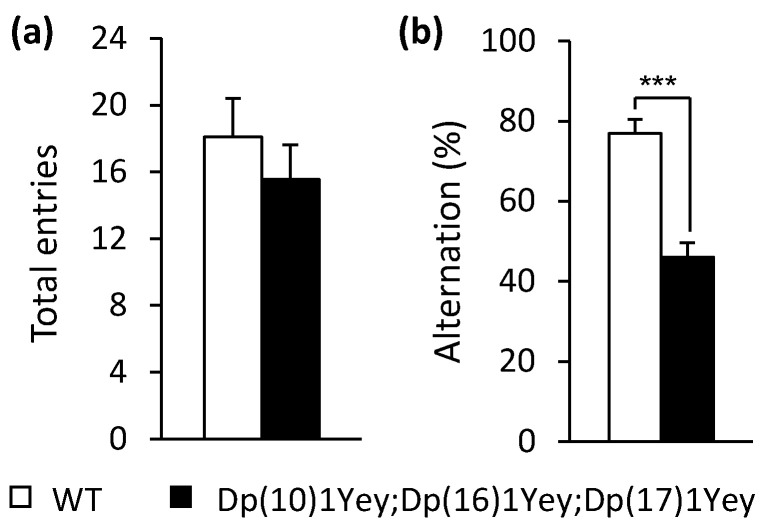
T-maze test: Dp(10)1Yey;Dp(16)1Yey;Dp(17)1Yey and the wild-type control mice were allowed to explore the arms of the T-maze for 10 min and each entry into each arm was recorded. (**a**) There was no difference in the average total entries between the two groups of mice (*p* = 0.43). (**b**) Alternation rate was calculated with the times of correct entries over total times of entries. The alternation rate of Dp(10)1Yey;Dp(16)1Yey;Dp(17)1Yey mice was significantly less than that of the wild-type control mice. *** *p* < 0.005.

Detailed results for the following four types of tests to examine the hippocampal function of the triple compound model are described below: the T-maze test, nesting test, contextual fear-conditioning test, and Morris water maze test. In the T-maze tests, the alternation rate of entry into the left or right arm is associated with reference and working memory [25]. In these tests, the average value for entries into the T-maze for Dp(10)1Yey;Dp(16)1Yey;Dp(17)1Yey mice was 15.55, which was similar to the value of 18.08 for the wild-type control mice (*p* = 0.43). However, the alternation rate of Dp(10)1Yey;Dp(16)1Yey;Dp(17)1Yey mice was significantly lower than that of the wild-type control mice (*p* < 0.001), thus indicating the triple compound mice had impaired hippocampal function (Figure 5).

**Figure 6:**
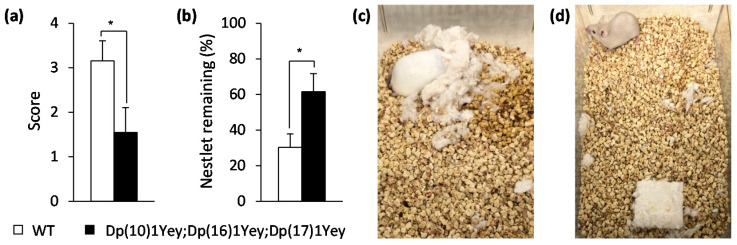
Nesting test: Dp(10)1Yey;Dp(16)1Yey;Dp(17)1Yey mice and the wild-type control mice were housed individually in a new cage with a pre-weighted Nestlet overnight. (**a**) The nest scores were 1.55 and 3.15 for the Dp(10)1Yey;Dp(16)1Yey;Dp(17)1Yey and wild-type control mice, respectively (*p* = 0.03). (**b**) The remaining Nestlet weighed 1.23 g for Dp(10)1Yey;Dp(16)1Yey;Dp(17)1Yey mice and 0.61 g for the wild-type control mice (*p* = 0.02). Dp(10)1Yey;Dp(16)1Yey;Dp(17)1Yey mice consistently scored worse and left more Nestlet behind than the wild-type control mice. (**c**) A representative picture of a nest built by a wild-type control mouse, which corresponded to a score of 4. (**d**) A representative picture of the Nestlet remaining in the cage of a Dp(10)1Yey;Dp(16)1Yey;Dp(17)1Yey mouse, which corresponded to a score of 1. * *p* < 0.05.

In the nesting test [26], Dp(10)1Yey;Dp(16)1Yey;Dp(17)1Yey mice showed a significantly lower score for nest building when compared to the wild-type control mice (*p* < 0.05), and these mice left significantly more unused Nestlet material compared to the wild-type control mice (*p* < 0.05) (Figure 6).

**Figure 7:**
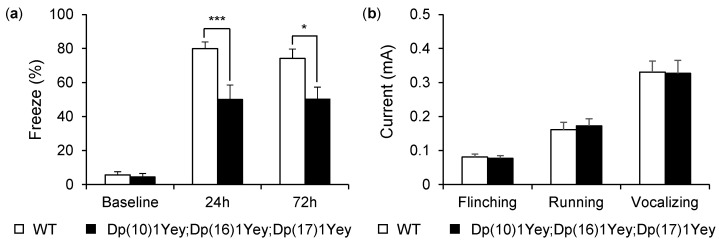
Contextual fear-condition test and foot-shock sensitivity test: (**a**) Dp(10)1Yey;Dp(16)1Yey;Dp(17)1Yey mice and the wild-type control mice were placed in the testing chamber for 2 min, and a 2-s foot-shock was delivered at the end of the period. Dp(10)1Yey;Dp(16)1Yey;Dp(17)1Yey mice and the wild-type control mice exhibited no significant difference at the baseline freezing level (*p* = 0.67). Then, mice were placed back into the chamber 24 and 72 h afterward. After 24 h, Dp(10)1Yey;Dp(16)1Yey;Dp(17)1Yey mice froze significantly less than the wild-type control mice (*p* = 0.003). After 72 h, Dp(10)1Yey;Dp(16)1Yey;Dp(17)1Yey mice still froze less than the wild-type control mice (*p* = 0.014). (**b**) A foot-shock sensitivity test was performed on both groups of mice. The minimum currents required to induce flinching, running, or vocalizing were similar for Dp(10)1Yey;Dp(16)1Yey;Dp(17)1Yey mice and the wild-type control mice. * *p* < 0.05; *** *p* < 0.005.

To examine the capacity for hippocampal-mediated contextual memory in mice, we performed the contextual fear-conditioning test [27,39]. Before the delivery of foot shocks, Dp(10)1Yey;Dp(16)1Yey;Dp(17)1Yey mice and their wild-type control mice showed a similar baseline freezing level (*p* > 0.05). After 24 h, both mutant mice and wild-type mice showed increasing freezing behavior upon return to the test chamber. However, Dp(10)1Yey;Dp(16)1Yey;Dp(17)1Yey mice froze significantly less than wild-type mice (*p* < 0.005). Then, 72 h after checking the baseline freezing level, the mice were returned to the original test chamber again, and we observed that the mutant mice still froze significantly less than the wild-type control mice (*p* < 0.05) (Figure 7a). To facilitate interpretation of the contextual fear-conditioning data, foot-shock sensitivity tests were performed, and these data showed that there was no difference between the two groups of mice in terms of the mean threshold of the current needed to elicit flinching, vocalizing, and running (*p* > 0.05) (Figure 7b).

**Figure 8:**
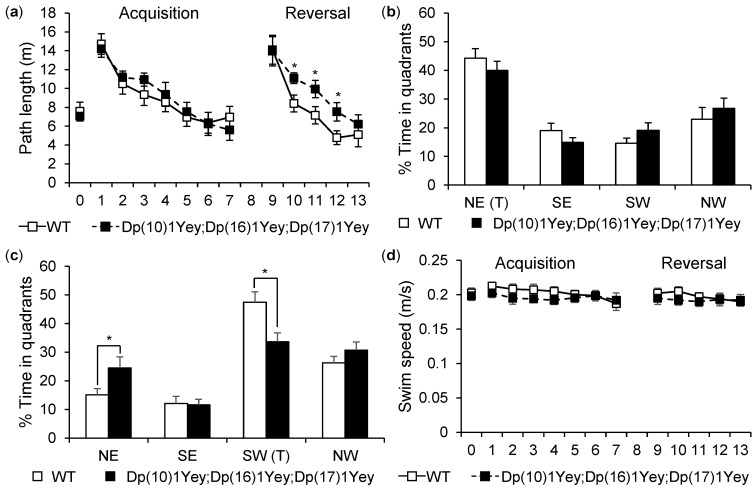
Morris water maze test: Dp(10)1Yey;Dp(16)1Yey;Dp(17)1Yey mice and the wild-type control mice were compared in a Morris water maze test. (**a**) During the hidden platform test from day 1 to 7, no differences in the path-lengths taken to reach the platform were observed between the mice carrying two different genotypes (* *p* > 0.05). During the reverse platform test from day 9 to 13, the path-lengths taken to reach the platform were longer for Dp(10)1Yey;Dp(16)1Yey;Dp(17)1Yey mice than those for the wild-type control mice (* *p* < 0.05). (**b**) In the probe test on day 8, no differences in the times spent in the target quadrant were observed between the mice carrying two different genotypes (*p* > 0.05). (**c**) In the reverse probe test on day 14, differences in the times spent in the target quadrant were observed between the mice carrying two different genotypes (* *p* < 0.05). (**d**) There was a small difference in swimming speed between the mice carrying two different genotypes (*p* < 0.05).

To examine hippocampal-mediated spatial learning and memory [27,28,29], we compared the performances of the Dp(10)1Yey;Dp(16)1Yey;Dp(17)1Yey mice and wild-type control mice in the Morris water maze test. In the hidden platform version, although the path-length needed for both groups to locate the platform significantly decreased following the training (*p* < 0.05), there were no significant differences detected between the mutant mice and the wild-type control mice (Figure 8a). In the probe test, the difference between the Dp(10)1Yey;Dp(16)1Yey;Dp(17)1Yey mice and wild-type control mice was not significant either (Figure 8b). However, when reversal learning was tested, Dp(10)1Yey;Dp(16)1Yey;Dp(17)1Yey mice exhibited significant deficits when compared with the wild-type control mice. Specifically, Dp(10)1Yey;Dp(16)1Yey;Dp(17)1Yey mice took a longer path-length to reach the platform, starting from the second day of reversal training trial (*p* < 0.05) (Figure 8a). Dp(10)1Yey;Dp(16)1Yey;Dp(17)1Yey mice also spent less time than the wild-type control mice in the target quadrant during the reversal probe tests (*p* < 0.05) (Figure 8c). Lastly, the mutant mice exhibited slower swimming speeds when compared with the wild-type control mice (*p* < 0.05) (Figure 8d).

The aforementioned behavioral results confirmed that Dp(10)1Yey;Dp(16)1Yey;Dp(17)1Yey mice identified via the coat color-facilitated strategy had significant deficits in hippocampal-mediated cognitive function.

## 4. Discussion

Down syndrome is one of the most complex human genetic disorders because it is associated with the triplication of all the genes on Hsa21. These triplicated genes not only affect phenotypes directly, but also interact among themselves and with disomic genes throughout genomes with consequences exhibited at the molecular, cellular, and organismal levels. Some of these interactions have been speculated to occur based on established knowledge, while others have been demonstrated experimentally [18,21,22,23,40,41,42]. To reflect these evolutionarily conserved interactions at all levels, it is desirable that all Hsa21 gene orthologs in an animal model are triplicated. If any of those genes remained at the disomic status in the model, this would create the possibility that some critical interactions may not have been mimicked. For this reason, the triple compound mouse model Dp(10)1Yey;Dp(16)1Yey;Dp(17)1Yey can serve as a reference model for DS in analyses of gene dosage effects because of its unique genotype.

The current study demonstrated that Dp(10)1Yey;Dp(16)1Yey;Dp(17)1Yey mice can be generated efficiently with the aid of coat colors. The value of 10.5% for the transmission ratio of the triple compound mice we obtained (Table 1) was lower than the value of 12.5% predicted by the Mendelian ratio. The magnitude of the reduction was approximately 17.9%, which was close to what we observed for the incidence rate of heart defects associated with the triple compound model [20].

Even though duplications, such as Dp(10)1Yey and Dp(16)1Yey, may facilitate sequence homology-based recombination, we have not detected this phenomenon among our mutants. If it has happened, the frequency of such a recombination must be very low according to the ratio of the genotypes of the progeny. If such an event does occur, the recombination will eliminate the targeted *Ty* gene as well as the duplication simultaneously, thus converting a mutant mouse carrying both *Ty* and Dp(10)1Yey or Dp(16)1Yey to a wild-type mouse. Therefore, regardless of whether or not sequence homology-based recombination takes place, *Ty* will co-segregate with Dp(10)1Yey or Dp(16)1Yey, unless the recombination is not sequence homology-based, which usually only occurs at an extremely low incidence rate.

On some occasions, the strain background of mice can have an important effect on a phenotype [43,44,45,46,47,48,49]. Because individuals with DS have different genetic backgrounds, it is desirable for mouse models of DS to also have a more mixed background, which better mimics the human condition, instead of an inbred or congenic strain background. If some DS phenotypes appear only in a model with an inbred or congenic background, these phenotypes may not represent mechanistically what happens in humans with DS. For these reasons, the mutant mice used in this study have been backcrossed to albino B6 for about two to three generations from the 129Sv background, instead of five generations, and this approach allowed us to avoid a background leaning forward congenic. This more mixed strain background could have reduced the incidence rate of heart defects slightly. The backcrossing process has resulted in different mixes of C57B6/J-*Tyrc*^-Brd^ and 129Sv backgrounds in individual mice. Because the wild-type C57B6/J-*Tyrc*^-Brd^ and 129Sv mice exhibit some differences in behaviors, the different contributions of C57B6/J-*Tyrc*^-Brd^ and 129Sv backgrounds may lead to variability of the phenotypes in individual Dp(10)1Yey;Dp(16)1Yey;Dp(17)1Yey mice beyond the impact of the triplications of the same Hsa21 gene orthologs. Sampling of a sufficient number of mice with different genotypes, as shown in the current study, reduce the strain background-associated variability of the phenotypes.

Our present study confirmed that the triple compound mutant mice generated with the coat color-facilitated strategy exhibited significant impairments in cognitive function. It is our expectation that the mutant mice generated with this coat color-facilitated strategy will exhibit many DS phenotypes detected in Dp(16)1Yey and Ts65Dn models with the advantage of reflecting the triplications of all Hsa21 gene orthologs in mice.

Recent establishment of TcMAC21 mice is another significant development in the efforts to model DS [11]. However, 14 Hsa21 genes were deleted in the transchromosome in the model and many of these deleted genes already have been implicated in important DS phenotypes [50,51,52,53,54,55,56,57]. In addition, incorporation of a human chromosome in mice may not precisely represent triplication of a part of the genomic material, as in human trisomy 21 in DS, because of the sequence differences between the syntenic regions in these two species. The regulatory elements influencing gene expressions may be different in humans and mice, which may lead to differential spatial and temporal expression patterns for the gene orthologs on mouse and human chromosomes [2,11]. Furthermore, some human proteins may not participate in normal oligomerization with mouse proteins; some human proteins may even function differently from mouse orthologous proteins [2], which may be considered as abnormal proteins in a transchromosomal mouse mutant. All these possibilities may complicate the interpretations of data generated from transchromosomal models.

Based on this work and other studies, all important animal models of DS have their advantages and disadvantages [11,14,58]. It may therefore be advisable to analyze different DS-associated phenotypic features and their underlying mechanisms in multiple animal models. For the same reason, different preclinical treatment strategies and their associated pharmacological mechanisms should be studied in multiple animal models. Such comparable analyses may yield surprising insights [58]. In conclusion, we can anticipate that the Dp(10)1Yey;Dp(16)1Yey;Dp(17)1Yey model generated via the much more efficient and cost-effective strategy described in this study will continue to play a critical role in DS research.

## Figures and Tables

**Table 1:** Analysis of the relationship between genotypes and coat colors among the progeny generated by crossing Dp(10)1Yey;Dp(16)1Yey mice to Dp(17)1Yey mice.

Genotype Group	(1)	(2)	(3)	(4)	(5)	(6)	(7)	(8)
Coat colors	Tan	Tan	Cream	Cream	Cream	Cream	White	White
Progeny #	23	25	31	23	28	24	34	31
Ratio	10.50%	11.42%	14.16%	10.50%	12.79%	10.96%	15.53%	14.16%

(1): Dp(10)1Yey;Dp(16)1Yey;Dp(17)1Yey; (2): Dp(10)1Yey;Dp(16)1Yey; (3): Dp(10)1Yey;Dp(17)1Yey; (4): Dp(16)1Yey;Dp(17)1Yey; (5): Dp(10)1Yey; (6): Dp(16)1Yey; (7): Dp(17)1Yey; (8): wild-type.

## Data Availability

The data presented in this study are available from the corresponding author upon reasonable request.
